# Intratumoral heterogeneity affects tumor regression and Ki67 proliferation index in perioperatively treated gastric carcinoma

**DOI:** 10.1038/s41416-022-02047-3

**Published:** 2022-11-08

**Authors:** Magnus Kock am Brink, Laura Sophie Dunst, Hans-Michael Behrens, Sandra Krüger, Thomas Becker, Christoph Röcken

**Affiliations:** 1 Department of Pathology, Christian-Albrechts-University, Kiel, Germany; 2 Department of General Surgery, Visceral, Thoracic, Transplantation and Pediatric Surgery, University Hospital Schleswig-Holstein, Kiel, Germany

**Keywords:** Translational research, Outcomes research

## Abstract

**Background:**

Intratumoral heterogeneity (ITH) is a major problem in gastric cancer (GC). We tested Ki67 and tumor regression for ITH after neoadjuvant/perioperative chemotherapy.

**Methods:**

429 paraffin blocks were obtained from 106 neoadjuvantly/perioperatively treated GCs (one to five blocks per case). Serial sections were stained with Masson’s trichrome, antibodies directed against cytokeratin and Ki67, and finally digitalized. Tumor regression and three different Ki67 proliferation indices (PI), i.e., maximum PI (KiH), minimum PI (KiL), and the difference between KiH/KiL (KiD) were obtained per block. Statistics were performed in a block-wise (all blocks irrespective of their case-origin) and case-wise manner.

**Results:**

Ki67 and tumor regression showed extensive ITH in our series (maximum ITH within a case: 31% to 85% for KiH; 4.5% to 95.6% for tumor regression). In addition, Ki67 was significantly associated with tumor regression (*p* < 0.001). Responders (<10% residual tumor, *p* = 0.016) exhibited prolonged survival. However, there was no significant survival benefit after cut-off values were increased ≥20% residual tumor mass. Ki67 remained without prognostic value.

**Conclusions:**

Digital image analysis in tumor regression evaluation might help overcome inter- and intraobserver variability and validate classification systems. Ki67 may serve as a sensitivity predictor for chemotherapy and an indicator of ITH.

## Introduction

Gastric cancer (GC) continues to pose a major health problem. With approximately 800,000 deaths annually, it is the third leading cause of cancer mortality worldwide [1]. Clinically and pathologically, GC is a highly heterogeneous disease with few options for targeted therapies [2] and modest response rates to conventional chemotherapy [3, 4]. By introducing an aggressive neoadjuvant/perioperative (hereinafter collectively referred to as neoadjuvant) chemotherapy regime for the treatment of resectable GC, survival significantly improved, yet prognosis of patients remains relatively poor mostly due to disease recurrence [5]. A large part of this problem stems from the marked intratumoral heterogeneity (ITH) of GC on the genetic and translational level [6–8]. As an example, ITH was described for several biomarkers including claudin 18.2, FGFR2, Her2/neu, Ki67 and PD-L1. Accurate assessment of a biomarker status is compromised, when only a few biopsy samples are available for testing [9–14], bearing the risk of false positive and false negative test results [10].

ITH stems from genetic and environmental constraints, with cancer being now considered an evolutionary disease with a complex and genetically distinct subclonal architecture [15]. ITH does not only affect biomarker expression but also impedes therapeutic response in a complex manner [16, 17]. Tumor regression, a biomarker used to assess therapeutic efficacy of neoadjuvant chemotherapy and predict patient survival [18, 19], is assessed by estimating the overall decrease in tumor mass [20]. This implies a homogeneous reaction to (radio-)chemotherapy, disregarding potential regional differences in regression, due to spatial ITH and consequent resistance to therapeutic agents. The same applies for proliferation. Tumor proliferation is often evaluated by determining the Ki67 proliferation index (PI) in a single selected tumor sample. The PI is thereby reduced to a single value supposedly representing the entire tumor. However, multiple authors demonstrated that Ki67 shows extensive ITH in untreated GCs, indicating regional differences in proliferation within the tumor [12, 21, 22]. To date, there is no data regarding ITH of Ki67 after neoadjuvant treatment, although neoadjuvant treatment has become standard of care in Europe [5].

We hypothesize that ITH in GC also applies to tumor regression and Ki67 PI after neoadjuvant chemotherapy. Furthermore, we were interested in the prognostic value of tumor regression and Ki67, and whether they correlate with each other. In order to test these hypotheses, we studied tumor regression and Ki67 PI in a series of 106 cases. Multiple samples per case were analyzed by Masson’s trichrome staining and immunostaining (cytokeratin and Ki67). Digital image analysis was used to assess tumor regression for each tumor block as well as Ki67 PI.

## Material and methods

### Study population

From the archive of the Department of Pathology, UKSH, Campus Kiel, we sought 108 patients who had undergone platinum based neoadjuvant (radio-)chemotherapy followed by either total or partial gastrectomy for adenocarcinoma of the stomach (distal) or esophago-gastric junction (proximal) between 2009 and 2018. The following patient characteristics were retrieved: type of surgery, age at diagnosis, gender, tumor size, tumor localization, tumor type, depth of invasion, residual tumor status, number of lymph nodes resected, and number of lymph nodes with metastases. Tumor regression was evaluated according to Becker et al. [20] into tumor regression grade (TRG) 1a (complete regression), TRG1b (<10% vital tumor cells), TRG2 (10% to 50% vital tumor cells) and TRG3 (>50% vital tumor cells).

Patients were included if an adenocarcinoma of the stomach or esophago-gastric junction was histologically confirmed. Patients were excluded if a tumor type other than adenocarcinoma was histologically identified. Each resected specimen had undergone gross sectioning and histological examination by trained and board-certified surgical pathologists. Date of patient death was obtained from the *Epidemiological Cancer Registry* of the state of Schleswig-Holstein, Germany. Follow-up data of those patients who were still alive were retrieved from hospital records and general practitioners. All patient data were pseudonymized after study inclusion.

### Histology

Tissue specimens were fixed in formalin and embedded in paraffin (FFPE). Deparaffinized whole mount tissue sections (WMTS) were stained with hematoxylin and eosin and Masson’s trichrome stain. Histological re-examination of primary tissue sections was carried out for all cases to assure all inclusion criteria were met. Tumors were classified according to Laurén [23] and re-examined by two surgical pathologists. pTNM-stage of all study patients was determined according to the 8th edition of the UICC guidelines [24]. The entire tumor bed of all resection specimens had been embedded and the number of paraffin blocks available depended on the size of the tumor bed. If more than five tumor-bearing blocks were available from a case (e.g., ≥20 tissue blocks), five representative tumor-bearing blocks were selected at random. If less than five blocks per case were present, all available tumor blocks were included in the study. In seven cases, residual tumor was present only in a single paraffin block although the entire tumor bed had been embedded.

### Immunohistochemistry

Immunohistochemistry was carried out with a rabbit monoclonal antibody directed against Ki67 (clone SP6, Abcam, Cambridge, United Kingdom) and a monoclonal antibody directed against pan-cytokeratin (#MS-343-P1, Epredia, Breda, Netherlands). Pretreatment for both antibodies was done with ER2 (Leica Biosystems, IL, USA) for 20 min. The Ki67 antibody was applied at a dilution of 1:300 and the pan-cytokeratin (PCK) antibody at a dilution of 1:200 using antibody diluent (Zytomed Systems, Berlin, Germany). Immunostaining was performed with the autostainer Bond™ Max System (Leica Microsystems GmbH, Wetzlar, Germany). The immunoreaction was visualized with the Bond™ Polymer Refine Detection Kit (brown labelling; Menarini Diagnostics, Berlin, Germany). Counterstaining was done with hematoxylin (Dr. K. Hollborn & Söhne GmbH&CoKG; Leipzig, Germany (#88663)).

### Digital image analysis

All immunohistochemically stained slides were digitalized at a maximal magnification of 40x using a Leica SCN400 Slide scanner (Leica Biosystems, Nussloch, Germany). Pixel-to-pixel distance measured 0.26 µm. The images were exported as SCN-files, which then were studied using a virtual microscopy program (VMP) that was previously used to examine digitalized WMTS [25]. The program featured the function to measure tumor area by using a polygonal-line drawing function as well as the ability to count cells by placing points of different colors. For the assessment of tumor regression, the line drawing function was used, while the Ki67 PI was determined by using the function to place differently colored points.

### Evaluation of Ki67

Two methods were used to determine the Ki67 proliferation index (PI). Besides the common procedure of evaluating only the region with the highest density (“hot spot”) of Ki67-positive tumor cells [12, 21], we additionally examined the region with the lowest density of Ki67-positive tumor cells. Thereby the highest (Ki67 high) and lowest (Ki67 low) possible Ki67 PI for each slide was determined and by measuring the difference (KiD) between Ki67 high (KiH) and Ki67 low (KiL) the span of proliferation was evaluated. For this purpose, the areas with the highest and lowest density of Ki67-positive tumor cells were delineated using the VMP-program. In each delineated area Ki67-positive tumor cells were marked with a red point, while Ki67-negative tumor cells were marked with a blue point. The PI of the KiH and the KiL were determined separately by the VMP. All placed points within a delineated polygon (either for KiH or KiL) were automatically detected by VMP and the Ki67 PI was determined by the ratio of all red dots (i.e., Ki67-positive tumor cells) to all placed dots of that polygon. Any nucleolear staining and/or predominant staining of the karyoplasm were evaluated as Ki67-positive [26–28]. No subdivision was made based on staining intensity. Cytoplasmic staining was evaluated as Ki67-negative. For both the KiH and the KiL up to 500 tumor cells were manually counted. A minimum of 100 tumor cells was necessary to be included into Ki67 evaluation, with each PI being evaluated on a minimum of 100 tumor cells. If more than 100 tumor cells were present but the tumor mass was only sufficient for one PI (e.g., 150 tumor cells), the evaluated PI was set as KiH.

### Assessment of tumor regression

**Fig. 1:**
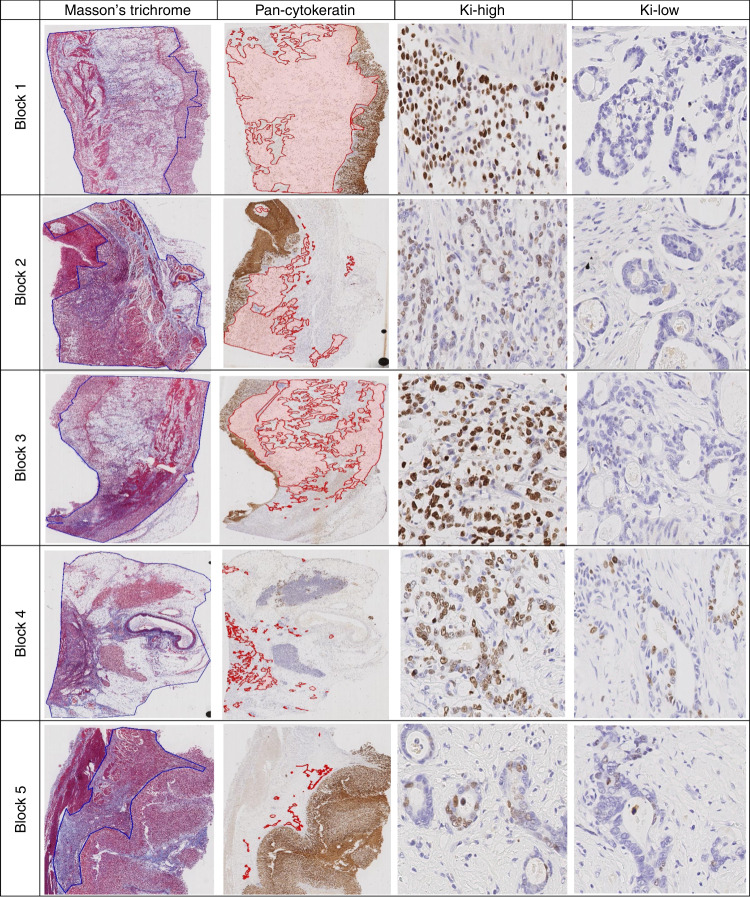
Illustration of intratumoral heterogeneity of Ki67 expression and tumor regression in the examplary case (Supplemental Fig. 1). Each row represents a seperate block of the same tumor. Tumor regression was evaluated as the ratio of residual (pan-cytokeratin) to former (Masson’s trichrome stain) tumor site. With increasing tumor regression, the hot spot Ki67 PI (KiH) decreased while the Ki67 PI at the lower proliferation end (KiL) increased (values in Supplemental Fig. 1). Ki67 images in 200-fold.

Tumor regression was determined using the PCK immunostaining and Masson’s trichome-stained slides. PCK immunostaining helped to identify tumor cells. Masson’s trichrome staining was used to delineate the former tumor bed [20]. Using the polygonal-line drawing function in both slides the respective area of residual tumor (PCK) and former tumor bed (Masson’s trichrome staining) were assessed according to a standardized protocol (Fig. 1). Tumor regression was then determined by calculating the ratio between residual PCK-positive tumor mass and former tumor bed and is hereafter referred to as tumor bed ratio (TBR).

### Study design (Supplemental Fig. 1)

Serial sections were cut from 1-5 paraffin blocks per case and forwarded to Masson’s trichrome stain and immunohistochemical staining with antibodies directed against Ki67 and PCK. To ensure sufficient tumor mass for the evaluation of Ki67 and tumor regression was present, all PCK immunostained slides were reviewed microscopically. If a minimum of fifty tumor cells were counted in the PCK stained slide, the slide was included into the study. From each paraffin block, we generated four parameters, i.e., KiH, KiL, KiD, and TBR. Each case consisted of one to five paraffin blocks, thus giving a minimum of 4 data points and a maximum of 20 data points per case. Subsequent data analysis was either based on a block-wise analysis, where data from each paraffin block were included or case-wise analysis, where 4 different parameters were determined, i.e., the maximum KiH and the minimum KiL of each case, the KiD between the maximum KiH and minimum KiH and the median of all TBRs (mTBR) measured for that case. Finally, the staining results (Ki67, PCK) were correlated with TBR, clinicopathological characteristics, and survival data.

### Statistical analysis

Statistical analyses were performed using SPSS 20.0 (IBM Corporation, New York, USA). mTBR and Ki67-values were dichotomized at their median. For the survival analyses, the mTBR was additionally divided into quartiles and further cut-off values for the mTBR were set at 10% and 20%. We used Fisher´s exact test for analysis of association between nominal and dichotomized variables. Ordinal scale variables were analysed using Kendall’s tau test. Overall (OS) and tumor-specific survival (TSS) was computed using the Kaplan-Meier method and compared by log-rank test to determine significance of differences between the survival curves. We assumed a significance level of 0.05. To compensate for the false discovery rate within the correlations, we applied the Simes (Benjamini-Hochberg) procedure (false discovery rate (FDR)-correction).

## Results

Initially, 108 cases fulfilled all study criteria with a total of 443 tumor blocks. After revision of the tissue specimens, 14 (3.2%) paraffin blocks and two cases were excluded from the study, due to an insufficient tumor mass. The final study collective consisted of 106 cases with 429 paraffin blocks (average 4.0 blocks/case) with five tumor-bearing paraffin blocks being available from 58 (54.7%) cases, four from 19 (17.9%) cases, three from 12 (11.3%) cases, two from 10 (9.4%) cases and in seven cases (6.6%) only a single tumor-bearing paraffin block was available.

### Evaluation of intratumoral heterogeneity

The TBR was evaluated in 429 WMTS and varied within a case and between cases (Fig. 1; Supplemental Fig. 2). The greatest difference was found in case 71 with a TBR ranging from 4.5% to 95.6%, and the lowest difference was observed in case 5 with a TBR ranging from 0.1% to 0.7%. No case showed identical TBRs in every WMTS studied. Thus, TBR showed substantial intra- and intertumoral heterogeneity, and did not differ between patients who received chemotherapy or chemoradiotherapy.

KiH was assessable in 394 WMTS, KiL, and KiD each in 376 WMTS. The median Ki67 PI was 61.5% (range: 1% to 99%) for KiH, 11.7% (range: 0% to 88%) for KiL and 40.4% (range: 0% to 96%) for KiD. As illustrated in Supplemental Fig. 2, KiH (red dot) and KiL (blue dot) varied substantially within each case, i.e., from block to block (=intratumoral heterogeneity) and between different cases (=intertumoral heterogeneity). Further, KiD showed high values (median: 40.4%) in the WMTS, indicating that Ki67 was heterogeneously expressed (different values were measured for KiH and KiL in the same WMTS) in each WMTS. Ki67 was not homogeneously distributed in the tumor blocks (similar values for KiH and KiL) but rather showed regional discrepancies. Thus, substantial intra- and intertumoral heterogeneity was also apparent for the three different Ki67-indices, i.e., KiH, KiL and KiD, and applied for both patients who received chemotherapy or chemoradiotherapy.

### Block-wise analysis

Next, we were interested to explore “block-wise” the correlation between KiH and TBR (*n* = 394 tumor blocks), as well as the correlation between KiL and KiD, respectively, and TBR (*n* = 376 tumor blocks). To perform this analysis, we dichotomized all TBR values of the cohort at the median (median TBR of block-wise analysis: 13.5%) into WMTS without signs of tumor response (=“non-responder blocks”; TBR above median) and WMTS with signs of tumor response (=“responder blocks”; TBR below median).

#### Ki67 and tumor bed ratio

Interestingly, all three Ki67-indices, i.e., KiH, KiL, and KiD correlated significantly with the TBR (*p* < 0.001). Compared to responder blocks, non-responder blocks showed higher Ki67-values for KiH and KiD. The opposite was observed for KiL. In responder tumor blocks, KiL values were significantly higher compared with non-responder blocks.

### Case-wise analysis

After conducting a “block-wise” analysis, we examined whether the same effects were also present when the data points (i.e., the KiH, KiL, KiD, and TBR values) were assigned to their respective case. For this purpose, we determined four “case-wise” parameters, which reflected the parameters (KiH, KiL, KiD, TBR) used in “block-wise” analysis and were derived from the data points given for each case. For each case we determined the mTBR (*n* = 106 cases; median of case-wise analysis: 12.2%; range: 0% to 86%), the maximum KiH (*n* = 99 cases; median: 75.8%; range: 4% to 99%), the minimum KiL (*n* = 97 cases; median: 4.0%; range 0% to 42%) and the KiD between maximum KiH/minimum KiL (*n* = 97 cases; median 66.8%; range: 2% to 98%). All parameters were dichotomized at their respective median for subsequent analysis. Similar to the “block-wise” analysis, the mTBR values were dichotomized into cases without signs of tumor response (=“non-responder cases”, mTBR above median) and cases with signs of tumor response (=“responder cases”; mTBR below median). All stated *p*-values were verified by Simes´ multiple testing procedure (complete data is shown in Table 1).

**Table 1:** Correlation of clinicopathological patient characteristics with tumor bed ratio, and Ki67 proliferation indices.

				Median tumor bed ratio dichotomized				Maximum KiH				Minimum KiL				KiD max KiH min KiL			
		Total		<median (responders)		≥median (non-responders)		<median		≥median		<median		≥median		<median		≥median	
		*n*	(%)	*n*	(%)	*n*	(%)	*n*	(%)	*n*	(%)	*n*	(%)	*n*	(%)	*n*	(%)	*n*	(%)
**Total**																			
**Gender**	**n p**^**(1)**^	106		106			1.000	99			**0.023**^**(4)**^	97			0.805	97			**0.047**^**(4)**^
Male		85	(80.2)	42	(49.4)	43	(50.6)	35	(44.3)	44	(55.7)	39	(50.6)	38	(49.4)	34	(44.2)	43	(55.8)
Female		21	(19.8)	11	(52.4)	10	(47.6)	15	(75.0)	5	(25.0)	11	(55.0)	9	(45.0)	14	(70.0)	6	(30.0)
**Age Group**	**n p**^**(1)**^	106		106			0.697	99			0.546	97			1.000	97			0.684
<64 Years		49	(46.2)	23	(46.9)	26	(53.1)	24	(54.5)	20	(45.5)	22	(51.2)	21	(48.8)	20	(46.5)	23	(53.5)
≥64 Years		57	(53.8)	30	(52.6)	27	(47.4)	26	(47.3)	29	(52.7)	28	(51.9)	26	(48.1)	28	(51.9)	26	(48.1)
**Localization**	**n p**^**(1)**^	106		106			1.000	99			**0.016**	97			0.825	97			**<0.001**
Proximal stomach		75	(70.8)	38	(50.7)	37	(49.3)	29	(42.0)	40	(58.0)	36	(52.9)	32	(47.1)	26	(38.2)	42	(61.8)
Distal stomach		31	(29.2)	15	(48.4)	16	(51.6)	21	(70.0)	9	(30.0)	14	(48.3)	15	(51.7)	22	(75.9)	7	(24.1)
**Laurén phenotype**	**n p**^**(1)**^	106		106			**0.042**^**(4)**^	99			**0.003**	97			0.989	97			**0.003**
Intestinal		53	(50.0)	33	(62.3)	20	(37.7)	26	(55.3)	21	(44.7)	24	(53.3)	21	(46.7)	24	(53.3)	21	(46.7)
Diffuse		23	(21.7)	8	(34.8)	15	(65.2)	17	(73.9)	6	(26.1)	11	(47.8)	12	(52.2)	17	(73.9)	6	(26.1)
Mixed		21	(19.8)	10	(47.6)	11	(52.4)	5	(25.0)	15	(75.0)	10	(50.0)	10	(50.0)	4	(20.0)	16	(80.0)
Unclassifiable		9	(8.5)	2	(22.2)	7	(77.8)	2	(22.2)	7	(77.8)	5	(55.6)	4	(44.4)	3	(33.3)	6	(66.7)
**ypT**	***n*** **p**^**(2)**^	106		106			**<0.001**	99			0.148	97			0.154	97			0.432
ypT1a/T1b		17	(16.0)	12	(70.6)	5	(29.4)	8	(66.7)	4	(33.3)	5	(41.7)	7	(58.3)	7	(58.3)	5	(41.7)
ypT2		16	(15.1)	12	(75.0)	4	(25.0)	10	(71.4)	4	(28.6)	5	(41.7)	7	(58.3)	9	(75.0)	3	(25.0)
ypT3		63	(59.4)	28	(44.4)	35	(55.6)	26	(41.3)	37	(58.7)	33	(52.4)	30	(47.6)	25	(39.7)	38	(60.3)
ypT4a/T4b		10	(9.4)	1	(10.0)	9	(90.0)	6	(60.0)	4	(40.0)	7	(70.0)	3	(30.0)	7	(70.0)	3	(30.0)
**ypN**	**n p**^**(2)**^	106		106			**<0.001**	99			0.095	97			0.084	97			0.133
ypN0		35	(33.0)	22	(62.9)	13	(37.1)	20	(64.5)	11	(35.5)	15	(50.0)	15	(50.0)	19	(63.3)	11	(36.7)
ypN1		23	(21.7)	16	(69.6)	7	(30.4)	9	(42.9)	12	(57.1)	5	(23.8)	16	(76.2)	7	(33.3)	14	(66.7)
ypN2		28	(26.4)	12	(42.9)	16	(57.1)	13	(48.1)	14	(51.9)	17	(63.0)	10	(37.0)	16	(59.3)	11	(40.7)
ypN3		20	(18.9)	3	(15.0)	17	(85.0)	8	(40.0)	12	(60.0)	13	(68.4)	6	(31.6)	6	(31.6)	13	(68.4)
**UICC stage**	**n p**^**(2)**^	106		106			**0.003**	99			**0.003**	97			0.087	97			**0.003**
IA/IB		17	(16.0)	11	(64.7)	6	(35.3)	8	(66.7)	4	(33.3)	6	(50.0)	6	(50.0)	7	(58.3)	5	(41.7)
IIA/IIB		16	(15.1)	13	(81.3)	3	(18.8)	12	(75.0)	4	(25.0)	3	(20.0)	12	(80.0)	13	(86.7)	2	(13.3)
IIIA/IIIB/IIIC		60	(56.6)	25	(41.7)	35	(58.3)	27	(46.6)	31	(53.4)	34	(58.6)	24	(41.4)	25	(43.1)	33	(56.9)
IV/IVA/IVB		13	(12.3)	4	(30.8)	9	(69.2)	3	(23.1)	10	(76.9)	7	(58.3)	5	(41.7)	3	(25.0)	9	(75.0)
**ypL**	**n p**^**(1)**^	106		106			**<0.001**	99			**<0.001**	97			0.828	97			**<0.001**
ypL0		74	(69.8)	46	(62.2)	28	(37.8)	44	(64.7)	24	(35.3)	35	(53.0)	31	(47.0)	41	(62.1)	25	(37.9)
ypL1		32	(30.2)	7	(21.9)	25	(78.1)	6	(19.4)	25	(80.6)	15	(48.4)	16	(51.6)	7	(22.6)	24	(77.4)
**ypV**	**n p**^**(1)**^	106		106			**<0.003**	99			0.318	97			1.000	97			0.487
ypV0		97	(91.5)	53	(54.6)	44	(45.4)	47	(52.2)	43	(47.8)	45	(51.1)	43	(48.9)	45	(51.1)	43	(48.9)
ypV1		9	(8.5)	0	(0.0)	9	(100.0)	3	(33.3)	6	(66.7)	5	(55.6)	4	(44.4)	3	(33.3)	6	(66.7)
**ypPn**	**n p**^**(1)**^	106		106			0.073	99			0.504	97			0.657	97			0.652
ypPn0		79	(74.5)	44	(55.7)	35	(44.3)	38	(52.8)	34	(47.2)	35	(50.0)	35	(50.0)	36	(51.4)	34	(48.6)
ypPn1		27	(25.5)	9	(33.3)	18	(66.7)	12	(44.4)	15	(55.6)	15	(55.6)	12	(44.4)	12	(44.4)	15	(55.6)
**R-status**	**n p**^**(1)**^	104		104			0.359	97			0.755	95			1.000	95			0.759
pR0		92	(88.5)	47	(51.1)	45	(48.9)	45	(52.3)	41	(47.7)	42	(50.0)	42	(50.0)	43	(51.2)	41	(48.8)
pR1		12	(11.5)	4	(33.3)	8	(66.7)	5	(45.5)	6	(54.5)	6	(54.5)	5	(45.5)	5	(45.5)	6	(54.5)
**Becker grading**	**n p**^**(2)**^	106		106			**<0.001**	99			**<0.001**	97			**0.012**	97			**<0.001**
1b		29	(27.4)	27	(93.1)	2	(6.9)	16	(72.7)	6	(27.3)	5	(25.0)	15	(75.0)	16	(80.0)	4	(20.0)
2		19	(17.9)	14	(73.7)	5	(26.3)	14	(73.7)	5	(26.3)	10	(52.6)	9	(47.4)	15	(78.9)	4	(21.1)
3		58	(54.7)	12	(20.7)	46	(79.3)	20	(34.5)	38	(65.5)	35	(60.3)	23	(39.7)	17	(29.3)	41	(70.7)
**mTBR**	**n p**^**(1)**^							99			**0.016**	97			**0.002**	97			**0.015**
<median (responder)								27	(58.7)	19	(41.3)	15	(34.1)	29	(65.9)	28	(63.6)	16	(36.4)
≥median (non-responder)								23	(43.4)	30	(56.6)	35	(66.0)	18	(34.0)	20	(37.7)	33	(62.3)
**Overall Survival [months]**	**p**^**(3)**^	96		96			**0.014**	90			0.823	88			0.811	88			0.372
Total/events/censored		96/52/44		46/21/25		50/31/19		44/26/18		46/24/22		45/24/21		43/25/18		43/24/19		45/25/20	
Median Survival		26.6 ± 3.5		32.2 ± 7.6		13.2 ± 5.2		24.6 ± 3.2		22.4 ± 5.0		22.8 ± 6.5		26.6 ± 3.3		26.6 ± 4.3		22.1 ± 7.4	
95% C.I.		[19.9 - 33.4]		[17.3 - 47.2]		[2.9 - 23.4]		[18.3 - 30.9]		[12.5 - 32.3]		[10.0 - 35.5]		[20.2 - 33.0]		[18.3 - 35.0]		[7.7 - 36.6]	
**Tumor Specific Survival [months]**	**p**^**(3)**^	96		96			**0.034**^**(4)**^	90			0.742	88			0.801	88			0.478
Total/events/censored		96/41/55		46/17/29		50/24/26		44/20/24		46/19/27		45/17/28		43/21/22		43/19/24		45/19/26	
Median Survival		30.9 ± 2.8		61.5 ± 27.9		22.1 ± 7.7		30.9 ± 8.8		32.0 ± 9.0		32.3 ± n.c.		27.3 ± 5.0		27.3 ± 8.9		26.7 ± 8.0	
95% C.I.		[25.3 - 36.4]		[6.9 - 116.1]		[7.0 - 37.3]		[13.6 - 48.1]		[14.4 - 49.7]		n.c.		[17.5 - 37.2]		[10.0 - 44.7]		[11.1 - 42.3]	

KiH: maximum Ki67 proliferation index maximum; KiL: minimum Ki67 proliferation index; KiD: the difference between KiH/KiH; n.c. not computed, (1) Fisher’s exact test; (2) Kendall’s tau test; (3) log-rank test; (4) not significant after multiple testing correction.

#### Ki67 and tumor bed ratio

While the minimum KiL and the KiD of each case showed a significant (*p* = 0.002; *p* = 0.015) association with the mTBR, no association for the maximum KiH (*p* = 0.16) was found. Analogous to the results of the block-wise analysis, KiL showed higher values in “responder cases” while KiD was higher in “non-responder cases”.

Using the Pearson correlation coefficient, no correlation was found between Ki67 index and age of paraffin blocks (Supplemental Fig. 3).

### Survival analysis

**Fig. 2:**
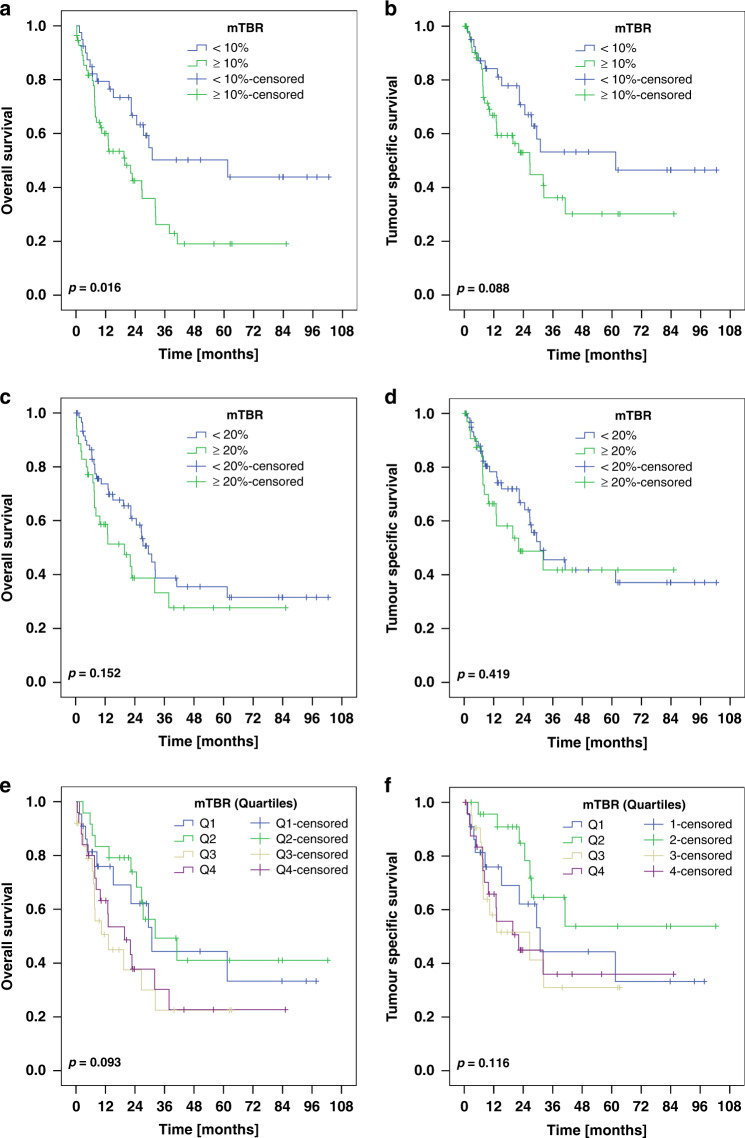
Kaplan-Meier plots for the whole cohort depicting patient survival. We performed three analyses setting the cut-off value for the median tumor bed ratio (mTBR) to 10% (**a, b**), to 20% (**c, d**) and additionally partitioned the cohort into quartiles (**e**, **f**). While patients with a mTBR below 10% exhibited prolonged survival (OS, *p* = 0.016), no prognostic benefit was shown at the cut-off value of 20%. Moreover, a finer grading into quartiles did not prove to be of prognostic relevance.

**Fig. 3:**
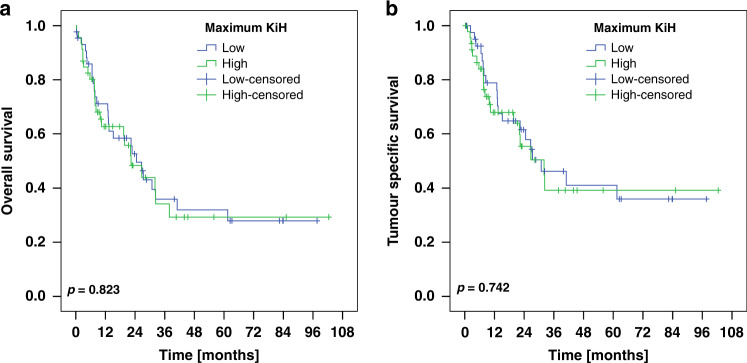
Patient survival depicted using Kaplan-Meier plots. Ki67 values were dichotomized at the median. No significant association with overall (**a**) or tumor-specific survival (**b**) was found for the maximum Ki67 hotspot proliferation index per case (maximum KiH).

mTBR was identified as a prognostic biomarker. After discovering prolonged survival for patients with a mTBR below 12.2% (median of the cohort; OS; *p* = 0.014; Supplemental Fig. 4), we set the cut-off value to 10% (=TRG1 according to Becker et al. [20]) with similar results (Fig. 2a*;* OS; *p* = 0.016). However, after a slight increase of the cut-off value (to 20%), no significant survival benefit was observed (Fig. 2c; OS; *p* = 0.152). The same applied for mTBR values being divided into quartiles (Fig. 2e, f; Table 2). With regard to Ki67, no prognostic significance was found for any of the Ki67 parameters (maximum KiH, minimum KiL, KiD maximum KiH/minimum KiL) (Fig. 3, only maximum KiH shown).

**Table 2:** Correlation of the tumor bed ratio with patient survival.

	Tumor bed ratio in quartiles							
	Quartile 1 (0–2%)		Quartile 2 (>2–12%)		Quartile 3 (>12–33%)		Quartile 4 (>33–100%)	
	*n*	(%)	*n*	(%)	*n*	(%)	*n*	(%)
**Overall Survival [months]**	96							0.093
Total/events/censored	22/10/12		24/11/13		25/15/10		25/16/9	
Median Survival [years]	30.9 ± 6.4		32.2 ± 10.7		13.2 ± 5.5		19.8 ± 6.4	
95% C.I.	[18.2–43.5]		[11.3–53.2]		[2.3–24.0]		[7.2–32.4]	
**Tumor Specific Survival [months]**	96							0.116
Total/events/censored	22/10/12		24/7/17		25/11/14		25/13/12	
Median Survival [years]	30.9 ± 6.4		n.c.		26.6 ± 12.8		22.1 ± 9.3	
95% C.I.	[18.2–43.5]		n.c.		[1.5–51.7]		[3.9–40.4]

### Correlation with clinicopathological patient characteristics

Several patient characteristics remained significantly associated with Ki67 and mTBR (Table 1). High values for the mTBR, the maximum KiH and the KiD parameter were significantly associated with advanced GCs (UICC III/IV; *p* = 0.003 for all three parameters) and lymphatic invasion (pL1; *p* < 0.001 for all three parameters). The maximum KiH and the KiD parameter were significantly higher in mixed and unclassified GCs than in intestinal and diffuse GCs (both *p* = 0.003). Proximal tumors were also associated with higher values for the maximum KiH and the KiD parameter (*p* = 0.016; *p* < 0.001). In addition, mTBR was significantly higher in large tumors (pT3/pT4; *p* < 0.001), in GCs with multiple lymph node metastasis (pN2/pN3; *p* < 0.001) and vascular invasion (pV1; *p* < 0.001). At last, all four parameters correlated significantly with the Becker TRG [20]. GC´s with a poor response to neoadjuvant chemotherapy (TRG 3) had higher values for the mTBR, the maximum KiH, and the KiD parameter (*p* < 0.001 for all three parameters). In contrast, high values for the minimum KiL parameter were found in GCs with a good response to neoadjuvant chemotherapy (TRG 1; *p* = 0.012).

## Discussion

Intratumoral genetic heterogeneity has moved into focus in recent years, due to its impact on diagnostics and treatment. Over time, the accumulation of random somatic driver (growth advantage) and passenger (neutral) mutations leads to the branching of genetically distinct tumor cell clusters known as subclones. Depending on whether a mutation is present in all tumor cells or only in a smaller subset of tumor cells, they are referred to as a trunk (clonal) or branch (subclonal) mutation. This process is highly dynamic and influenced by numerous factors like the microenvironment, spatial constraints, intersubclonal competition, or chemotherapy [17, 29–31]. As a result, the tumor landscape is shaped by the coexistence of multiple subclones, each containing a unique genetic composition and biomarker expression pattern.

### Intratumoral heterogeneity of Ki67 is maintained after chemotherapy

Ki67 was first described in 1983 by Gerdes et al. and is frequently used to measure proliferative activity in a variety of malignancies [32]. Ki67 is strictly bound to the active phases of proliferation (G_1_, S, G_2_ phase, and mitosis) and is not expressed during cellular resting (G_0_) [26, 27]. Several authors have presented evidence for ITH of Ki67 in untreated GCs, however, ITH was mostly determined visually without quantification [12, 21, 22]. Böger et al. and Ramires et al. both reported heterogeneous expression patterns of Ki67 with either focal clustering (“clonal”) or zonal differences in superficial and deeper compartments [12, 22]. Ramires et al. partially objectified their observation in 29 out of 43 cases by determining a PI in the surface and in deeper layers, with significant differences between both measuring points [22]. An interesting study by Müller et al. was the first study to systematically assess ITH of Ki67 by determining multiple Ki67 PIs per tumor block, each being of different value (i.e., hot spot: 51.3%; invasion front: 37.2%; randomly chosen areas: 34.2%) [21]. However, assessment of biomarker heterogeneity should not be tied to fixed points of measurement (i.e., invasion front), but rather focus on differences in expression. We suggest that to quantify Ki67 heterogeneity, the highest (KiH) and lowest (KiL) Ki67 PI per sample should be determined. By assessing the difference (KiD) between KiH and KiL, an objective measure of Ki67 heterogeneity is obtained. In addition to ITH, substantial intertumoral heterogeneity of Ki67 expression has been reported (Müller et al.: 3% to 92% [21], Böger et al.: 2% to 99% [12]), which we can confirm in our cohort (maximum KiH range: 4% to 99%). Based on the available data, our study supports the contention that inter- and intratumoral heterogeneity of Ki67 expression is maintained after neoadjuvant treatment, although information on Ki67 status before treatment is lacking in our cohort. Further, comparison with previous studies regarding Ki67 ITH is limited, due to differences in methodological aspects and chemotherapy status (treated vs. untreated).

### Tumor regression exhibits extensive intratumoral heterogeneity

Tumor regression is either evaluated by the ratio of viable tumor cells to former tumor site, as conducted in this study, or by the ratio of viable tumor cells to fibrotic tissue scarring. All classifications have in common that tumor regression is divided into different grades based on the overall decrease in tumor mass [33]. However, it might be misleading to assume that tumor regression is a homogeneous process. Besides anatomic localization or histological phenotype [18, 34], the microenvironment plays a crucial role in tumor response to treatment by promoting hypoxia, inflammation, fibrosis, and a resulting favorable metabolic state for the tumor cells [35]. Tumor microenvironment however displays extensive ITH in immune contexture, vascularity distribution, and cancer-associated fibroblasts, impairing therapeutic efficacy and indicating that tumor regression varies regionally within the tumor [36]. From a molecular and genetic standpoint, several mechanisms like uptake carriers, export pumps, or DNA repair systems influence the response of tumor cells to chemotherapeutic agents and can be present simultaneously in the tumor, acting in a synergistic manner [35]. Although decisive evidence for ITH in resistance mechanisms is missing, interindividual variances in DNA damage repair pathways [37] and genetic polymorphisms were described for GC [38]. Thus, it can be assumed that due to the mutational and genetic heterogeneity of subclonal architectures, there is a high probability that different resistance mechanisms to treatment will develop in different parts of the tumor prior to chemotherapy (e.g., stem mutation in most subclones or branch mutation in a few subclones), potentially leading to heterogeneous regression patterns within the tumor [6, 31]. To our knowledge, our study is the first to provide evidence for substantial ITH in tumor regression in GC (Fig. 1; Supplemental Fig. 2). However, it is only to speculate that intratumoral genetic heterogeneity is responsible for the variances in tumor regression observed in this study, thus further studies investigating whole sectioned tumors and their mutational composition in resistance mechanisms are needed.

### Neoadjuvant chemotherapy inhibits rapidly proliferating tumor cells while promoting subclonal selection

**Fig. 4:**
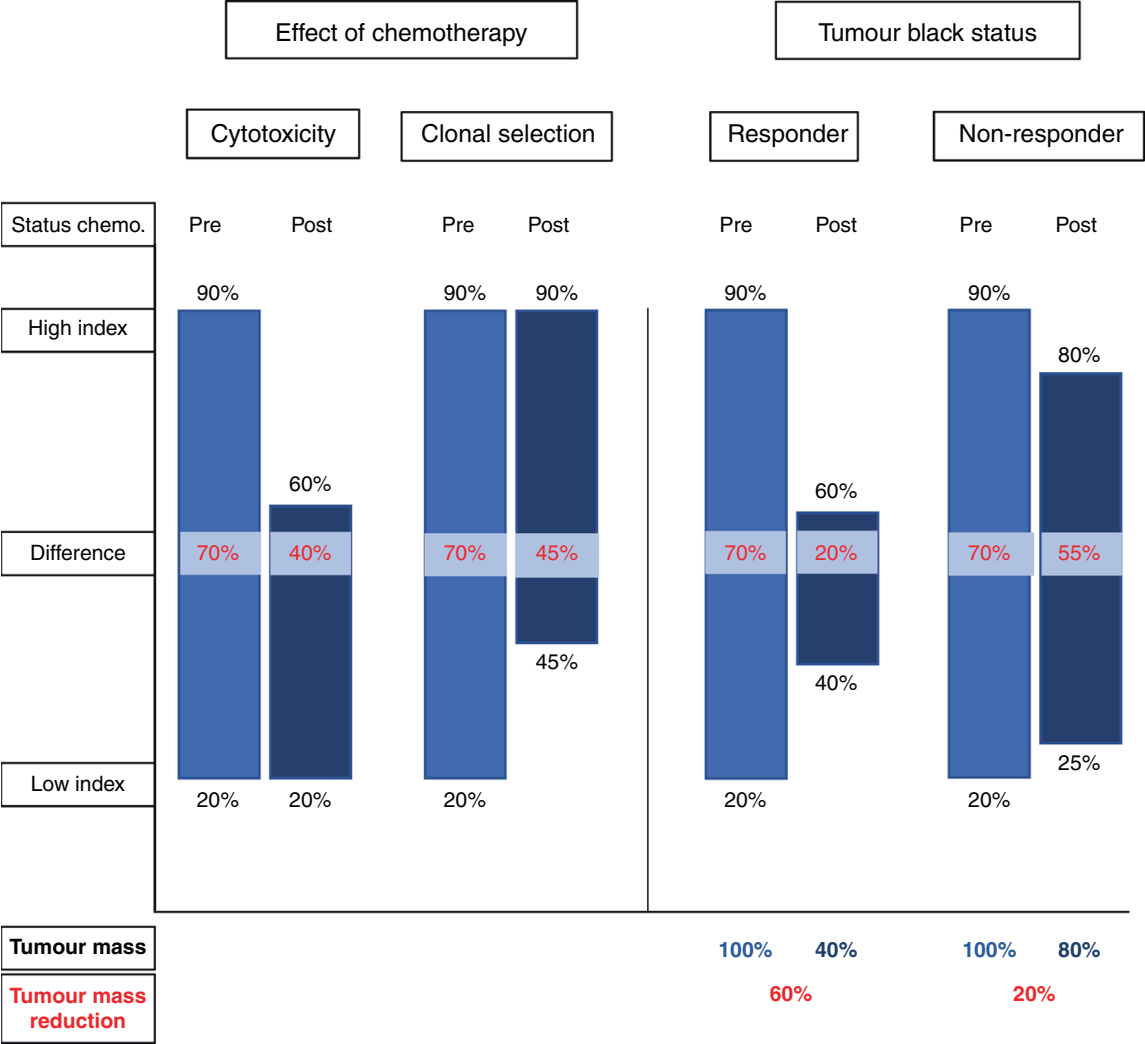
This schematic model illustrates the putative effect of neoadjuvant chemotherapy on Ki67 proliferation index (PI) and tumor regression. Each pair of bars represents the Ki67 PI prior to neoadjuvant chemotherapy (light blue; expected value) and post-chemotherapy (dark blue; measured value). Ki67 high index (KiH), Ki67 low index (KiL), the difference (KiD) between KiH/KiL and tumor mass reduction were specified for each bar. Two main effects were observed (left). The decline in the KiH is interpreted as a result of the cytotoxic effect chemotherapy has on proliferating, chemo-sensitive subclones. The incline in the KiL can be attributed to selection pressure exerted by neoadjuvant chemotherapy on clonal diversity. Prior to neoadjuvant chemotherapy, chemo-resistant subclones were inhibited in their growth by better adapted (“fitter”), chemo-sensitive subclones. Following selection caused by neoadjuvant therapy, the surviving (chemo-resistant) subclones were able to exploit their growth advantage (increase in proliferation). Both effects were observed in tumor blocks with a large reduction of tumor mass (right; responder). Chemo-sensitive subclones perished with chemo-resistant subclones being selected through neoadjuvant chemotherapy. In contrast, in tumor blocks where chemo-resistant subclones accounted for a large proportion of the tumor mass (non-responder), only minor changes in KiH and KiL were observed.

Chemotherapy plays a major role in the treatment of GC, with impact on both proliferation and tumor regression (Fig. 4). Fast proliferating tissues, like tumor cells, are the primary subject to chemotherapeutic agents. Therefore, it is not surprising that Ki67 was reported to be significantly lower in a cohort treated with neoadjuvant chemotherapy [39], with high Ki67 values prior to therapy indicating a good response [40]. Simultaneously, it was reported that with increasing pathological response, Ki67 values declined [39]. This is consistent with our results, however, no information on Ki67 status, and thus Ki67 dynamics, was available for our cohort before treatment (KiH and TBR, *p* < 0.001; maximum KiH and Becker TRG [20], *p* < 0.001; Table 1). Despite methodical differences in the assessment of Ki67 and tumor regression, these observations indicate that Ki67 may serve as a predictor for chemotherapy sensitivity. Chemotherapy may not only affect rapidly proliferating cells but also exert selective pressure on subclonal architectures [31, 41, 42]. Chemotherapy can act as a bottleneck, positively selecting treatment-resistant subclones, while eradicating chemotherapy-sensitive subclones. This has been reported for other malignancies, with our data indicating evidence for this in GC [43, 44]. We observed across all tumor blocks and at the level of the individual case that tumors with a good response to treatment showed increased proliferative activity at the lower end of the proliferation spectrum compared to tumors with a poor response (block-wise: KiL and TBR, *p* < 0.001; case-wise: minimum KiL and mTBR, *p* = 0.002). It can be assumed that a strong reduction of tumor mass simultaneously leads to an increased demise of tumor cell clones, leaving more room for expansion processes of selected, therapy-resistant subclones. This may be attributed to the omission of inhibitory influences by other subclones (competition) [31] or by selection of subclones with a favorable status in proliferation-associated mutations. Since there is a time lag between surgery and the last cycle of neoadjuvant chemotherapy, the increased proliferation can also be explained by the acquisition of new, subclonal mutations [41]. However, subsequent proliferation is not necessarily linked to a detectable selection process of better-adapted tumor clones. A study by Kreso et al. on colorectal cancer reported that within a uniform genetic lineage, individual tumor cells vary in their growth dynamics and response to therapy. Not all tumor cells contribute equally to tumor proliferation, but there exist tumor cells that oscillate between dormancy and proliferative activity or are held in reserve. This heterogeneity in single-cell functional behavior can trigger post-treatment propagation, without any substantial selection process [45].

### Ki67 is a putative biomarker for intratumoral genetic heterogeneity

Based on spatial differences in Ki67 PI, Böger et al. and Müller et al. suggested that Ki67 may also be a surrogate marker of genetic ITH [12, 21] and hence subclonal architecture. In our cohort, advanced tumor regression was associated significantly with a decreased KiD (*p* = 0.015). Chemotherapy eradicates treatment-sensitive subclones, which reduces the tumor mass and temporarily overall tumor heterogeneity [41]. In colorectal cancer neoadjuvant chemotherapy decreases genetic ITH, due to a massive demise of tumor cell subclones [44]. To the opposite, we provide evidence that a higher KiD is significantly associated with an unfavorable response to chemotherapy. In breast cancer, genetic diversity did not substantially change pre- and post-treatment in poor responders. However, cases with complete pathological response harbored low pretreatment genetic diversity [46]. Thus, tumor regression is linked to genetic diversity. Interestingly, we found higher KiDs in proximal and mixed-type tumors, and lower KiDs in distal and diffuse type tumors. According to The Cancer Genome Atlas, proximal tumors are often characterized by chromosomal instability with higher rates of focal amplifications in proliferation-associated receptor tyrosine kinases like EGFR and mutations in *TP53* [47]. Fittingly, ITH in *TP53* inactivation mechanisms were reported by Wong et al. [48], where *TP53* seems to be closely-linked to increased somatic mutations [49] and Ki67 expression [50]. Genomically stable GCs in contrast, are mostly found in the distal stomach and are highly associated with the diffuse type according to Laurén [47]. In this matter, Wong et al. reported that the extent of clonality in diffuse-type GC is significantly lower compared to intestinal-type GC [48]. In summary, our study supports the contention that KiD indicates genetic ITH and that Ki67 may be used as surrogate marker of ITH.

### Ki67 proliferation index is unsuitable as a prognostic biomarker after neoadjuvant treatment, despite accounting for intratumoral heterogeneity of Ki67 expression

Ki67 is used as prognostic biomarker in e.g., breast cancer [51] or lymphomas [52]. In contrast, its prognostic value in GC remains controversial. Although a recent meta-analysis demonstrated that high Ki67 values are significantly associated with an unfavorable overall survival [50], several studies, including our present study, found no significant association between Ki67 and patient prognosis [12, 21, 50]. In regard to neoadjuvantly treated GCs, only one study by Wu et al. provided quality data on Ki67, indicating that high Ki67 PI levels are prognostically unfavorable. However, areas of interest were selected randomly (obviously ignoring the risk of sampling error due to ITH; see above), and the cut-off value was set relatively low (11.9%) [39]. The opposing results regarding patient survival partly stem from lack of standardization in type and concentration of antibodies used, immunohistochemical staining protocol, number of tumor cells evaluated, cut-off values used, and type and number of tumor samples assessed [50]. A presumably even greater influence arises from the fact that the hotspot Ki67 PI (i.e., KiH) is usually determined in a single sample (i.e., block-wise), neglecting the impact of ITH on the entire tumor area (i.e., case-wise). Recently, Böger et al. demonstrated that tissue micro arrays significantly underestimate Ki67 PI compared to WMTS (on average 16.9%) [12]. Considering the substantial variation in Ki67 PI between individual regions of the same tumor found in this study (KiH range example case: 85% to 31%, Fig.1; Supplemental Fig. 2), our results provide striking evidence that Ki67 ITH is even more pervasive than previously assumed. Given the high risk of sampling bias due to ITH and the inconsistent evaluation criteria, Ki67 seems unsuitable to reliably predict patient prognosis. However, if future studies plan to investigate Ki67 as a prognostic biomarker, we propose that the evaluation should be based on precisely defined criteria in multiple samples per case to ensure accurate assessment.

### Confirmation of the 10% cut-off value while highlighting the pitfalls of eyeballing in tumor regression assessment

Prognostic biomarkers are essential for tailoring oncologic treatment, yet few biomarkers besides the TNM-classification of the UICC [24] are frequently used for GC. Tumor regression is often utilized to quantify response to treatment, but despite becoming increasingly important as a biomarker over the past decade, its prognostic role remains controversial. A recent meta-analysis revealed that tumor regression is significantly associated with superior survival [53]. To the contrary, Schmidt et al. found no association between TRG and prognosis in a series of 850 GCs [54]. Another problem is the different cut-off values of the classification systems, which lead to contradictory findings on which classification best predicts patient survival [55–58]. This is further aggravated by profound intra- and interobserver variability due to subjective eyeballing in tumor regression evaluation [59]. Although complete tumor regression is generally associated with the best patient outcome, there is little consensus regarding prognostic value in partial or subtotal regression. Despite these uncertainties, several studies came to the conclusion, that a TBR < 10% is significantly associated with superior survival, which we also confirmed, now using digital image analysis [18, 19, 60]. On the contrary, increasing the cut-off value to 20% or dividing into quartiles showed no statistical significance in our cohort. (Fig. 2c–f). Thus, even minor increases in measured tumor regression significantly impacted its prognostic value. Given the profound ITH in tumor regression (Fig. 1, Supplemental Fig. 2), pathologists are at constant risk of misevaluating the true extent of tumor regression. Hence, we urgently propose that tumor regression must be evaluated using digital image analysis with precisely defined evaluation parameters to minimize observer variability and eyeballing. Moreover, our approach might be of use to validate the different classification systems and their prognostic benefit and may even propagate a two-tiered scoring system (responder < 10% vs. non-responder >10%). However, our approach needs further confirmation in a larger cohort with, ideally, the entire tumor bed being evaluated.

### Limitations

Our study has several limitations. First, the subclonal architecture is likely complex, three-dimensional and a two-dimensional assessment in WMTS still carries the risk of a sampling error. Assumptions on subclonal architecture and its impact on proliferation and tumor regression are somewhat hypothetical. Second, tumor regression may not have been fully captured given that only a limited number of samples were assessed per case instead of the entire tumor. However, the study design primarily aimed to proof our hypotheses of ITH and not to assess the tumor regression for the entire tumor bed. Third, tumor cell proliferation is a highly dynamic process and evaluation of Ki67 only provides a snapshot in time. Furthermore, despite expression of Ki67, a tumor cell may not fully complete the cell cycle [28]. Thus, Ki67 may not be the best biomarker for tumor cell proliferation and patient prognosis. Forth, chemotherapy complicated Ki67 assessment in patients with substantial tumor regression, due to few spatially separated tumor cells being present. Finally, our methodological approach in Ki67 evaluation might be unsuitable for daily practice, due to the immense effort required in staining, digitalization, evaluation, and exploitation of automated digital image analyses. However, artificial intelligence may help to improve assessment of tumor regression and proliferation indices in the future.

### Conclusions and future directions

Our study may have implications for daily practice in the evaluation of tumor regression and provides useful information on tumor behavior after chemotherapy. We are the first to provide evidence for ITH of Ki67 and tumor regression after chemotherapy. Intratumoral genetic heterogeneity appears to be associated with tumor regression and proliferation, with Ki67 possibly indicating subclonal diversity, most likely at the level of proliferation-associated mutations. Although Ki67 seems to be unsuitable as a reliable prognostic biomarker, it may have the potential to serve as an indicator of sensitivity to chemotherapy and subclonal selection. However, this needs to be confirmed by further studies examining the relationship between Ki67 and genetic composition in multiple samples, ideally before and after treatment. In addition, we can confirm the prognostic value of tumor regression in a two-tier system that distinguishes between responders and non-responders [18, 20]. Moreover, our study provides striking evidence that the prognostic value of tumor regression is restricted to a very narrow “diagnostic window” leaving little room for rating errors, which easily reach beyond ±10% when eyeballing is applied. Our approach ensures a more objective evaluation of tumor regression. Our findings might be of use to help overcome intra- and interobserver variability in future studies and validate different TRG classifications.

## Supplementary information

Supplemental figure legends

Suppl. Figure 1

Suppl. Figure 2

Suppl. Figure 3

Suppl. Figure 4

Checklist

## Data Availability

The data presented in this study are available on request from the corresponding author.
