# Supplementary material for: Intratumoral heterogeneity affects tumor regression and Ki67 proliferation index in perioperatively treated gastric carcinoma
Source: Br J Cancer. 2022 Nov 8;128(2):375–86. doi: 10.1038/s41416-022-02047-3 (PMC9902476; doi:10.1038/s41416-022-02047-3)
Supplement: Supplementary file 1 — Supplemental figure legends [file 41416_2022_2047_MOESM1_ESM.docx]

**Intratumoral heterogeneity affects tumor regression and Ki67 proliferation index in perioperatively treated gastric carcinoma**

Magnus Kock am Brink,^1^ Laura Sophie Dunst,^1^ Hans-Michael Behrens,^1^

Sandra Krüger,^1^ Thomas Becker,^2^ Christoph Röcken^1^

1 Department of Pathology, Christian-Albrechts-University, Kiel, Germany

2 Department of General Surgery, Visceral, Thoracic, Transplantation and Pediatric Surgery, University Hospital Schleswig-Holstein, Kiel, Germany

**Supplemental figure legends**

**Supplemental Figure 1: Study design**

**Supplemental Figure 2:** The visualization of the data revealed inter- and intratumoral heterogeneity (ITH) in tumor regression and Ki67 expression. The cases are given as numbers on the x-axis in chronological order (case 1 is from 2009; case 106 from 2018). All data points were arranged orthogonally above their respective case on the x-axis, with the percentages for the TBR tumor bed ratio (TBR) values and Ki67 proliferation indices (PI) being plotted on the y-axis. Each colored shape represented a datapoint for either the TBR (purple), the Ki67 high index (KiH; red), the Ki67 low index (KiL; blue) or the difference (KiD; green) between KiH/KiL. The heterogeneity in tumor regression was shown by TBR values of a case being distributed over a range of percentages **(A).** ITH of Ki67 was complex. First, high values were measured for KiD, indicating that Ki67 was heterogeneously expressed in the tumor blocks **(C).** Second, the values for KiH and KiL were different in each block of the same case. Therefore, accurate assessment of Ki67 PI using only one tumor block could be affected by selection bias **(B).**

**Supplemental Figure 3:** The date of paraffin embedding (=age of the paraffin blocks) did not correlate with Ki67 high index (KiH; **A**; Pearson correlation coefficient: 0.003, p=0.955) or the Ki67 low index (KiL; **B**; Pearson correlation coefficient: -0.053, p=0.305). All data points were arranged orthogonally above their respective case on the x-axis, with the date of paraffin embedding being plotted on the y-axis.

**Supplemental Figure 4:** Kaplan-Meier plots for the whole cohort depicting patient survival. Median tumor bed ratio (mTBR) values were dichotomized at the median (Median 12.2%), with significant prolonged survival for patients with mTBR values below 12.2% (overall survival, p=0.014; tumor specific survival, p=0.034). However, results for tumor specific survival did not remain significant after multiple testing correction was applied.
