# Supplementary figures and images for: Intratumoral heterogeneity affects tumor regression and Ki67 proliferation index in perioperatively treated gastric carcinoma
Source: Br J Cancer. 2022 Nov 8;128(2):375–86. doi: 10.1038/s41416-022-02047-3 (PMC9902476; doi:10.1038/s41416-022-02047-3)

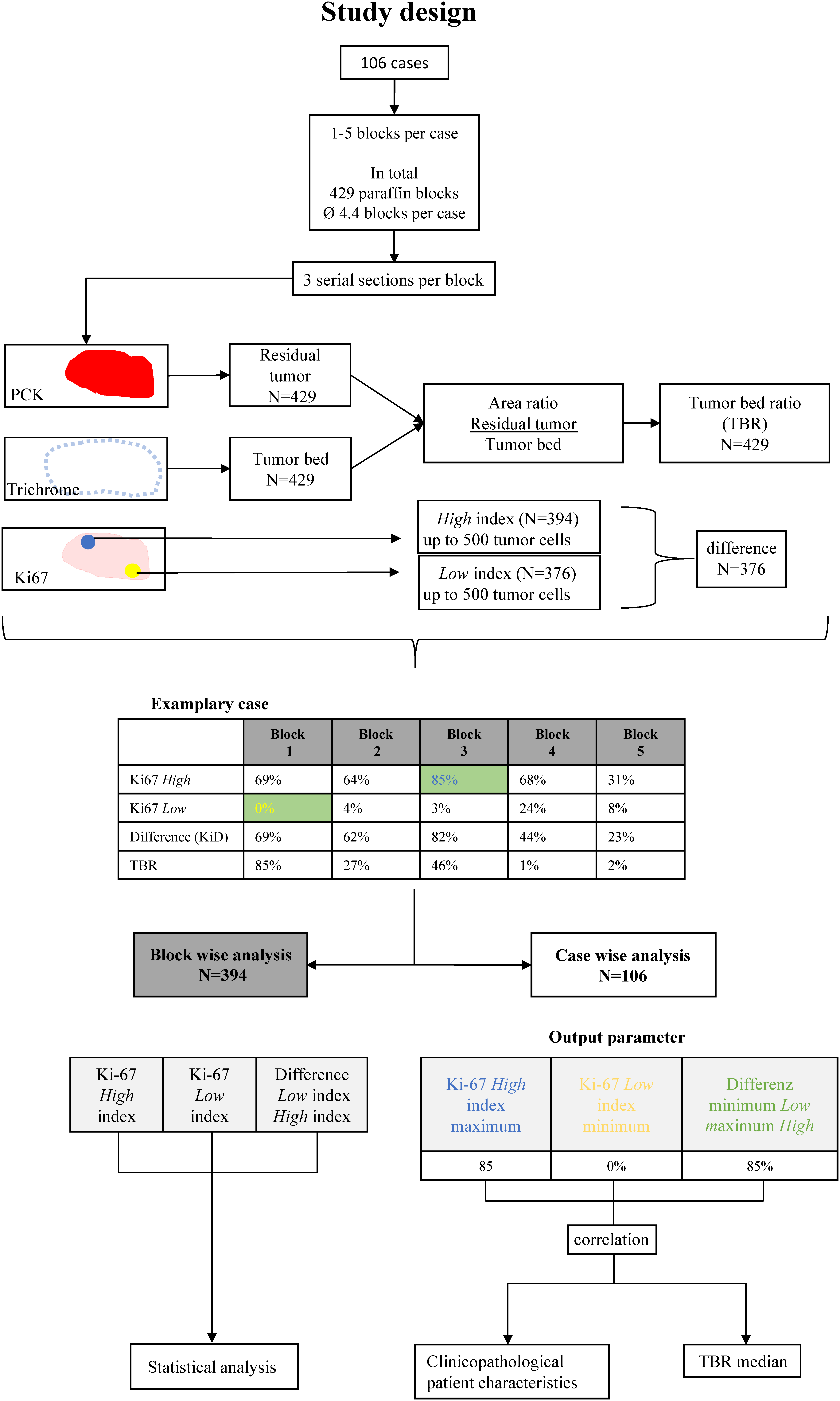

Supplement: Supplementary file 2 — Suppl. Figure 1 [file 41416_2022_2047_MOESM2_ESM.tif]

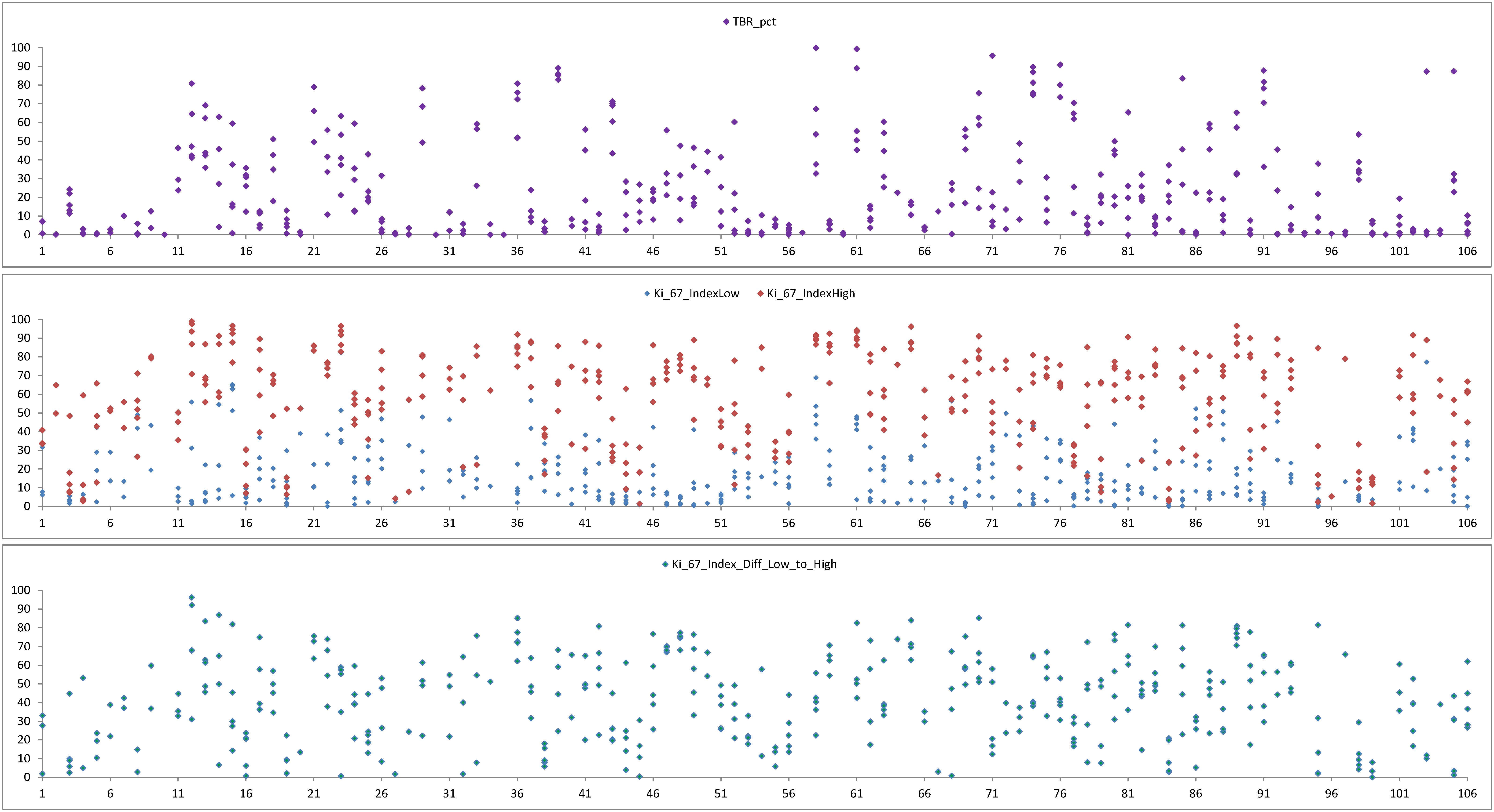

Supplement: Supplementary file 3 — Suppl. Figure 2 [file 41416_2022_2047_MOESM3_ESM.tif]

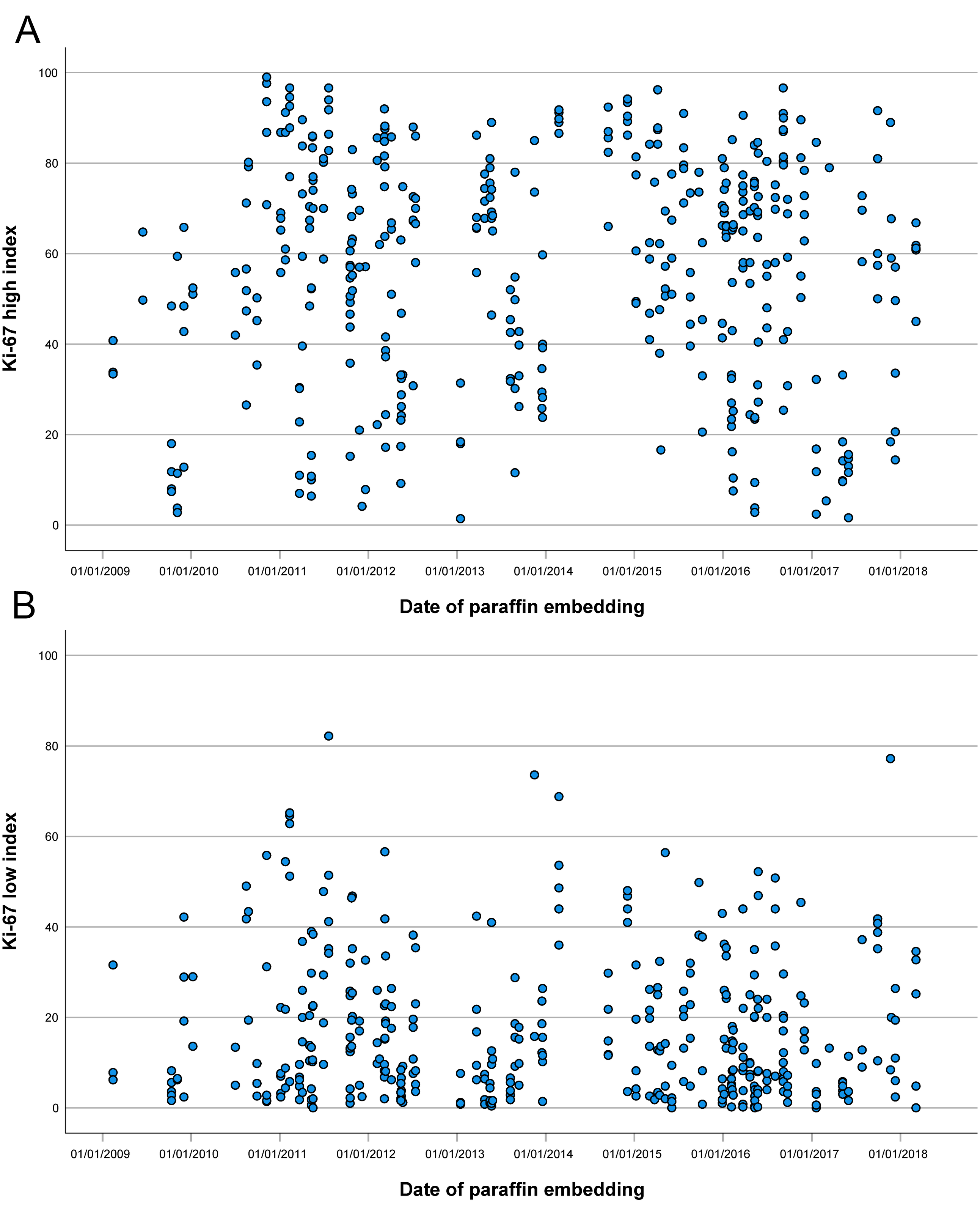

Supplement: Supplementary file 4 — Suppl. Figure 3 [file 41416_2022_2047_MOESM4_ESM.tif]

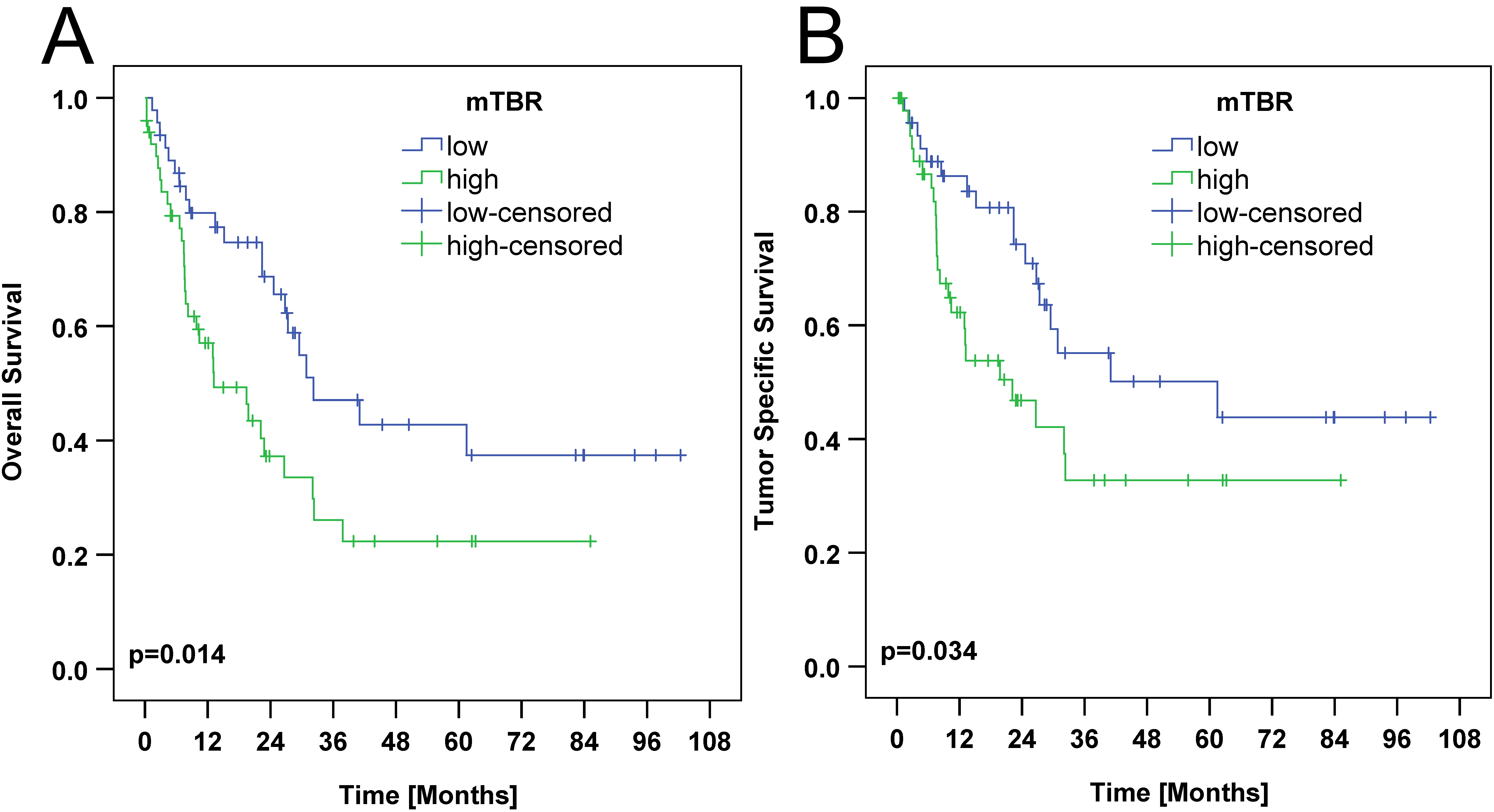

Supplement: Supplementary file 5 — Suppl. Figure 4 [file 41416_2022_2047_MOESM5_ESM.tif]
